# Zinc, Carnosine, and Neurodegenerative Diseases

**DOI:** 10.3390/nu10020147

**Published:** 2018-01-29

**Authors:** Masahiro Kawahara, Ken-ichiro Tanaka, Midori Kato-Negishi

**Affiliations:** Department of Bio-Analytical Chemistry, Faculty of Pharmacy, Musashino University, 1-1-20 Shinmachi, Nishitokyo-shi, Tokyo 202-8585, Japan; k-tana@musashino-u.ac.jp (K.-i.T.); mnegishi@musashino-u.ac.jp (M.K.-N.)

**Keywords:** zinc, copper, synapse, amyloid, apoptosis, ER stress, food analysis

## Abstract

Zinc (Zn) is abundantly present in the brain, and accumulates in the synaptic vesicles. Synaptic Zn is released with neuronal excitation, and plays essential roles in learning and memory. Increasing evidence suggests that the disruption of Zn homeostasis is involved in various neurodegenerative diseases including Alzheimer’s disease, a vascular type of dementia, and prion diseases. Our and other numerous studies suggest that carnosine (β-alanyl histidine) is protective against these neurodegenerative diseases. Carnosine is an endogenous dipeptide abundantly present in the skeletal muscles and in the brain, and has numerous beneficial effects such as antioxidant, metal chelating, anti-crosslinking, and anti-glycation activities. The complex of carnosine and Zn, termed polaprezinc, is widely used for Zn supplementation therapy and for the treatment of ulcers. Here, we review the link between Zn and these neurodegenerative diseases, and focus on the neuroprotective effects of carnosine. We also discuss the carnosine level in various foodstuffs and beneficial effects of dietary supplementation of carnosine.

## 1. Introduction

Zinc (Zn) is an essential trace element abundantly present after iron (Fe). It is a co-factor of more than 300 enzymes or metalloproteins, and plays a critical role in many functions including cell division, immune system, protein synthesis, and DNA synthesis [1,2]. Increasing evidence suggests that Zn acts as a second messenger in various biological systems, similar to calcium (Ca) [3]. Zn reportedly binds with a Zn-binding motif or metal-responsive element of about 10% of all proteins and regulates their levels of expression by recent bioinformatics research of the human genome [4]. 

Given these crucial functions in humans, Zn deficiency causes various adverse effects [5,6,7,8]. Zn deficiency in childhood causes the retardation of mental and physical development, learning disabilities, dwarfism, and dysfunction of the immunological system in humans. Because Zn is essential for olfaction and taste, Zn deficiency leads to learning, taste, and olfactory disorders in adults. Moreover, Zn deficiency is related to levels of depression and stress. 

It was recommended that the estimated average requirement (EAR) of Zn is 12 mg/day for adult males and 9 mg/day for adult females by the Japanese Ministry of Health, Labour and Welfare. Although daily intake of Zn is estimated to be 10–15 mg, many patients suffer from a mild Zn deficiency because Zn intake and absorption are distinctive. The bioavailability of Zn is influenced by many food constituents such as phytates and fibers in plants, which form poorly soluble complexes with Zn and inhibit its gastrointestinal absorption [9]. Yasuda and Tsutui reported that approximately 20% or more of the elderly and children in Japan suffers from Zn deficiency [10]. The World Health Organization reported that 1.4% (0.8 million) of deaths worldwide are attributed to Zn deficiency [11]. Therefore, supplementation is important for the prevention and treatment of Zn deficiency. Zn supplementation therapy is used in the treatment of pressure ulcers, measles, and taste disorders [12,13,14]. A complex of Zn and carnosine (β-alanyl histidine), termed polaprezinc, is widely used for this purpose [15]. Polaprezinc is also used for protecting the mucosa against ulcerations and for the treatment of Helicobacter pylori-associated gastritis [16,17]. 

**Figure 1:**
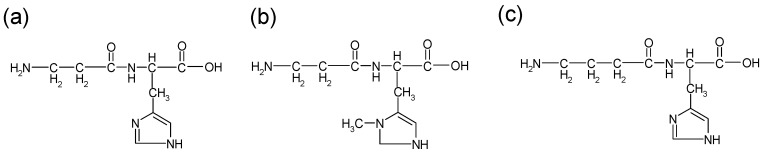
Structures of carnosine and its analogues: (**a**) carnosine (**b**) anserine (**c**) homocarnosine.

Carnosine is an endogenou dipeptide [18]. Carnosine is small and water-soluble. Carnosine and its analogues (homocarnosine and anserine) (Figure 1) exist in many organisms such as birds, fish, and mammals, including humans. It is abundantly present in skeletal muscles, but is also observed in the stomach, kidneys, cardiac muscle, and brain. Thus, daily foods such as meats or fish contain considerable amounts of carnosine. 

**Figure 2:**
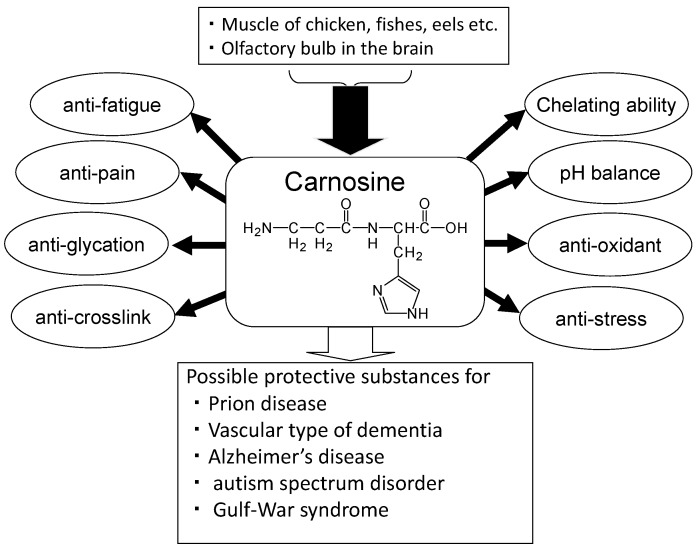
Beneficial effects of carnosine.

Carnosine has various advantageous characteristics, such as anti-glycation, anti-stress, and antioxidant properties, hydroxyl radical scavenging, maintenance of pH-balance, and chelation of metals including divalent zinc ion (Zn^2+^) and bivalent copper ion (Cu^2+^) (Figure 2) [19]. Carnosine is one of the most abundant small-molecule compounds in skeletal muscle, with concentrations similar to those of creatine and adenosine triphosphate (ATP). Carnosine contributes to the physicochemical buffering of lactate caused by exercise in skeletal muscles and has anti-fatigue effects. It is possible that carnosine contributes to the regulation of Zn availability in the brain [9]. Ours and other numerous studies indicate that carnosine is neuroprotective against various neurodegenerative diseases such as Alzheimer’s disease (AD) [20], the vascular type of senile dementia (VD) [21], prion diseases [22], autism spectrum disorder [23], and Gulf War syndrome [24]. Furthermore, supplementation therapy with carnosine and anserine is reported to be effective for improving cognitive impairment in the elderly [25]. 

Here, with a focus on the neuroprotective functions of carnosine, we review the link between Zn and neurodegenerative diseases, and investigate the neuroprotective roles of carnosine in terms of nutrients as a drug for these neurodegenerative diseases. We also discuss the levels of carnosine in foodstuffs based on our developed convenient quantitative analysis method using high performance liquid chromatography (HPLC).

## 2. Roles of Zinc in the Brain

Zn is abundantly present in the testes, muscle, liver, and brain tissues, and total Zn content is approximately 2 g. Zn is accumulated in the hippocampus, amygdala, cerebral cortex, thalamus, and olfactory cortex in the brain [26]. Zn is estimated to be present as 70–90 ppm (~20 µM) in the hippocampus [27]. Zn in the brain binds to metalloproteins or enzymes; however, approximately 10% or more Zn is stored in the presynaptic vesicles of glutamatergic excitatory neurons as free zinc ions (Zn^2+^). During neuronal excitation, the chelatable Zn^2+^ is secreted into synaptic clefts from vesicles with glutamate. The secreted Zn reportedly regulates the overall excitability of the brain by binding with various neurotransmitter receptors such as *N*-methyl-d-aspartate (NMDA)-type glutamate receptors, amino-3-hydroxy-5-methyl-4-isoxazolepropionic acid (AMPA)-type glutamate receptors, γ-aminobutyric acid (GABA) receptors, and glycine receptors [28]. Ueno et al. reported that secreted Zn^2+^ modulates spatio-temporal information in the hippocampus [29]. Thus, the secreted Zn is essential for synaptic plasticity, information processing, and memory formation. Indeed, Zn is reported to be essential for the induction of long-term potentiation (LTP) in the mossy fiber, a form of synaptic information storage [30]. It is possible that secreted Zn^2+^ has neuromodulators roles. Since Zn has been shown to generate neural inhibition, Zn can modulate the activity of the neighboring synapses by diffusing into synaptic clefts to the adjacent synapses in a distance-dependent manner. A similar phenomenon was reported as ‘lateral inhibition’, which caused the contrast of signals underlying the mechanism of synaptic plasticity [31].

Similar to Zn^2+^, Cu^2+^ was also reported to exist in synaptic vesicles. During neuronal excitation, Cu^2+^ is secreted into the synaptic cleft [32]. The secreted Cu^2+^ modulates neuronal excitability by binding to various receptors, including the GABA receptor, AMPA-type glutamate receptor, and NMDA-type glutamate receptor. The size of the synaptic cleft is estimated to be 20 nm in height and 120 nm in width, and is composed of ~1% of the total extracellular volume in the brain [33]. Considering its small size, it is more than plausible that the levels of neurotransmitters or metals are high in the synapse. Indeed, the concentration of glutamate is estimated to reach the mM range after neuronal depolarization in the synaptic cleft. Although the level of Zn in cerebrospinal fluid (CSF) is less than 1 μM, its level in the synaptic clefts is reported to be 1–100 μM [34]; meanwhile, the level of Cu^2+^ in the synaptic clefts is reported to be 2–15 μM [35]. Since Zn^2+^ and Cu^2+^ compete for entry into the body or for many binding proteins, it is possible that excess Zn can also lead to Cu dyshomeostasis.

**Figure 3:**
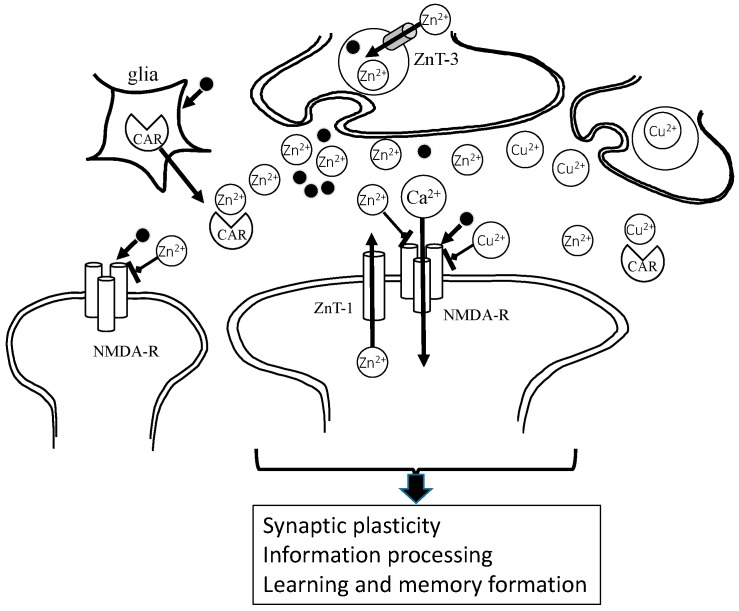
Under physiological conditions, Zn^2+^ and glutamate are released from presynaptic vesicles, and inhibit NMDA-type glutamate receptors (NMDA-Rs) and regulate other receptors. Zn^2+^ spilled over into the neighboring synapse, modulating excitability. It is possible that Zn is essential in the maintenance of synaptic plasticity and the formation of memory. Cu^2+^ is also secreted and acts in a similar manner to Zn^2+^. Carnosine binds to Zn^2+^, as well as Cu^2+^, and regulates these concentrations at the synapse. Zn^2+^: divalent zinc ion; Cu^2+^: bivalent copper ion; Ca^2+^: divalent calcium ion; CAR: carnosine; ZnT-1: zinc transporter 1, • glutamate.

Carnosine is reported to be synthesized in astrocytes and oligodendrocytes. It exists in olfactory bulb neurons and in glial cells, and is secreted from glial cells into the synaptic cleft [36]. Thus, it is highly possible that carnosine regulates Zn and Cu homeostasis in synaptic clefts. The hypothetical roles of Zn^2+^ and Cu^2+^ in the synapse are displayed in Figure 3.

Zn homeostasis is regulated by three factors, besides carnosine; metallothioneins, Zn transporters (ZnT), and Zrt-, Irt-like protein (ZIP) Zn transporters [37,38]. Metallothioneins are ubiquitous metal-binding proteins composed of 68 amino acids. These proteins possess 20 cysteine residues, and bind seven metal atoms including Cu, cadmium (Cd), and Zn. Among three types of metallothioneins: MT-1, MT-2, and MT-3, MT-3 is mainly observed in the central nervous system. However, MT-1 and MT-2 generally exist in the whole body.

There are 14 types of ZnTs in mammals. ZnTs decrease intracellular Zn by facilitating Zn efflux from cells. They are encoded with the solute carrier (*SLC30*) gene family. ZnT-1 has a pivotal role in Zn efflux and is involved in protection from excess Zn. ZnT-1 and ZnT-3 are co-localized with chelatable Zn in the brain. ZnT-3 transports Zn into synaptic vesicles, and maintains high Zn concentrations in these vesicles. 

ZIP Zn transporters transport Zn from extracellular compartments to those which are intracellular and increase cytosolic Zn. ZIP Zn transporters are encoded by fourteen *SLC39* genes. ZIP transporters are also located in the membranes of the Golgi apparatus or the endoplasmic reticulum (ER), and regulate Zn in subcellular organelles. Genetic defects of Zn transporter mutations produce severe diseases such as Ehlers-Danlos syndrome [39]. 

Furthermore, the excess or deficiency of Zn, namely, the dyshomeostasis of Zn in the brain, is considered to have relationships with the pathogenesis of several neurodegenerative diseases including AD, VD, prion diseases, and amyotrophic lateral sclerosis (ALS) [40,41,42,43].

## 3. Zinc, Carnosine and Alzheimer’s Disease

### 3.1. The Amyloid Hypothesis

In Japan, elderly adults aged more than 75 years represented 10% of the total population in 2013. Therefore, approximately 4 million people are affected by senile dementia and this is a number that continues to grow annually. 

**Figure 4:**
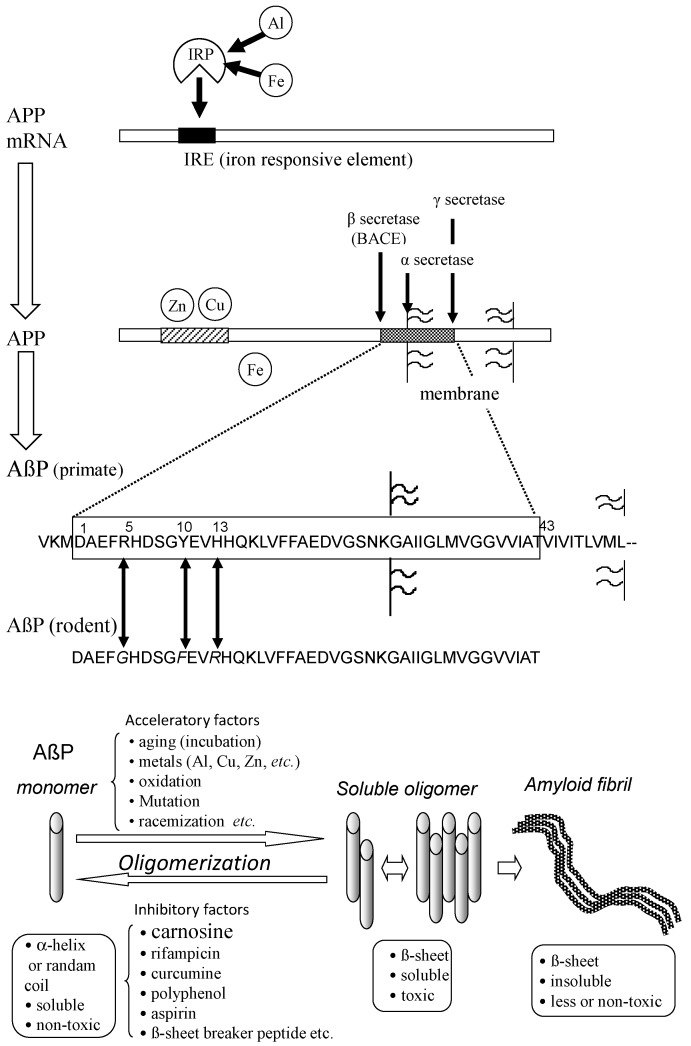
Secretion and oligomerization of AβP. AβP is secreted from APP by β-secretase (BACE) and by γ-secretase. The expression of APP is regulated by Fe and may be influenced by Al. APP also binds to Cu and Zn. Three amino acids (Arg^5^, Tyr^10^, and His^13^) of human AβP are substituted in rodent AβP. Under aging conditions or in the presence of acceleratory factors, monomeric AβP with random or α-helix structures self-aggregates and forms several types of oligomers (SDS-soluble oligomers, amyloid β-derived diffusible ligands, globulomers, protofibrils) before finally forming insoluble aggregates (amyloid fibrils). The monomeric and fibril aggregates are relatively nontoxic; however, oligomeric soluble AβPs are toxic. APP: amyloid precursor protein; mRNA: messenger ribonucleic acid; AβP: β-amyloid protein; Al: aluminum; Fe: iron; IRP: iron regulatory protein.

Most senile dementia is divided into AD, VD, and dementia with Lewy bodies (DLB). AD accounts for more than half the cases of senile dementia. Although AD was first reported in 1906, the number of AD patients was estimated to be more than 5 million in the U.S. in 2016. AD is characterized by the deposition termed senile plaques and neurofibrillary tangles (NFTs). The selective loss of synapses and neurons in the hippocampal and cerebral cortical regions is also observed [44]. The major component of NFTs is phosphorylated tau protein, and that of senile plaques is β-amyloid protein (AβP). The primary factor of AD pathogenesis remains controversial. The accumulation of tau protein and/or the degeneration of cholinergic neurons might occur during the pathogenesis. However, the idea of the amyloid cascade hypothesis, which suggests that the accumulation of AβP and the consequent neurodegeneration play a central role in AD is supported by many researchers [45,46]. AβP is a small peptide composed of 39–43 amino acid residues. AβP is secreted from a large precursor protein (amyloid precursor protein; APP) in the N-terminal by β-secretase (β-site APP cleaving enzyme; BACE), which is followed by the intra-membrane cleavage of its C-terminal by γ-secretase. The truncated AβPs, such as AβP (1−40), the first 40 amino acid residues, or AβP (1–42) are produced by the different C-terminal cleavage of APP (Figure 4). Secreted AβP is generally degraded by specific proteases such as neprilysin. It was reported that APP mutations and AβP metabolism are associated with AD from genetic studies of early-onset cases of familial AD indicated in [47]. It was also revealed that mutations in the presenilin genes account for the majority of cases of early-onset familial AD. Presenilins are reported to be one of the γ-secretases. Their mutations also influence the production of truncated AβP and its neurotoxicity. 

In 1990, Yankner et al. found that AβP (1–40) caused the toxicity of cultured rat hippocampal neurons [48]. Although these findings were controversial, the neurotoxicity of AβP was demonstrated to be influenced by its oligomerization and subsequent conformational change [49]. AβP has a tendency to self-aggregate into oligomers. AβP exists as a monomeric protein when freshly prepared and dissolved in an aqueous solution, and exhibits a random coil structure. However, AβP forms aggregates (oligomers) after incubation at 37 °C for several days (aging). The oligomer AβP possess β-pleated sheet structures and finally forms insoluble aggregates (amyloid fibrils). Neurotoxicity of AβP was reported to be enhanced during the aging process [50]. 

### 3.2. Metals and Amyloid

AβP is reportedly secreted in the CSF of non-dementia aged individuals, as well as young individuals [51]. Therefore, not only the amount of AβP, acceleratory factors, or inhibitory factors of its oligomerization may be essential in AD pathogenesis. AβP oligomerization is influenced by peptide concentrations, pH, solvent composition, and temperature. The oxidation, mutation, and racemization of AβP can affect it [52]. Zn and other trace elements such as aluminum (Al), Cu, and iron (Fe) are important accelerating factors. The amino acid sequences of humans and rodents AβP are similar, and rodent AβP is different from primate AβP by only three amino acids (Arg^5^, Tyr^10^, and His^13^). However, the accumulation of AβP is rarely observed in the rodent brains. Indeed, rodent AβP has less of a tendency to aggregate compared with primate AβP in vitro [53]. Interestingly, all of these three amino acids have the ability to bind metals. Bush et al. found that Zn induced the oligomerization of AβP, even the low concentrations (300 nM) [54]. They also reported that Cu remarkably enhanced the AβP aggregation [55]. Zn binds to three histidine residues (His^6^, His^13^, and His^14^) and/or to carboxyl group of Asp^1^ of AβP [56]. 

However, the oligomerization of AβP by metals is still controversial. The morphologies of AβP oligomers with Al, Cu, Fe, and Zn are quite different [57]. Metals such as Al, Cu, Fe, and Zn alter the oligomerization and toxicity of AβP in a different manner [58]. Cu-oligomerized AβP is more toxic compared with Zn-oligomerized AβP [59]. We found that Al caused more marked oligomerization than other metals, such as Zn, Cu, Fe, and cadmium (Cd) [60]. Furthermore, Al-aggregated AβPs bind tightly to the surfaces of cultured neurons and form fibrillary deposits several days after exposure, compared to Zn-aggregated AβPs.

Meanwhile, Zn can attenuate AβP-induced neurotoxicity and contributes to AD as a protector [40]. Various adverse effects after AβP exposure are reported, such as the induction of cytokines, the induction of ER stress, the production of reactive oxygen species, and the abnormal increase of intracellular calcium levels ([Ca^2+^]_i_) [61]. Although these effects may interact with each other, the disruption of Ca^2+^ homeostasis could be the primary adverse event of AβP neurotoxicity given that Ca^2+^ is involved in numerous cellular functions [62]. Arispe et al. first demonstrated that AβP forms pore-like channel structures on artificial lipid bilayers, which are permeable to Ca^2+^ and other cations, multilevel, voltage-independent, and long-lasting [63]. We found that AβP forms pore-like channels on neuronal membranes [64], and demonstrated that AβP caused the increase of intracellular Ca^2+^ levels of cultured neurons using fura-2 Ca^2+^ imaging [65]. Based on these results, the ‘amyloid channel hypothesis’ was demonstrated; which suggests that the direct incorporation of AβPs on neuronal membranes and the subsequent increase of intracellular Ca^2+^ through the amyloid channels might be the primary event in AβP neurotoxicity [66]. AβP might share the similar mechanism underlying the toxicity of various antimicrobial or antifungal peptides that also exhibit channel-forming activity and cell toxicity in this respect [67]. We and other researchers found that the channel activity was inhibited by the exposure to Zn^2+^, and recovered by *o*-phenanthroline, a Zn chelator [64,68]. Since His residues are exposed to inner surfaces of amyloid channels, Zn can bind to these His residues and protect neurons from AβP-induced Ca dyshomeostasis [69]. Therefore, the role of Zn in the pathogenesis of AD is still controversial and Zn may act as a contributor of AD pathogenesis, as well as a protector. In this context, Zn might play a role like Janus, the ancient Roman god of doorways, who is depicted with two different faces [40].

Moreover, APP is a metal binding protein that has two Cu and/or Zn binding domains in its N-terminal. APP possesses the ability to reduce Cu^2+^ to Cu^+^. Cu and Zn influence the expression and processing of APP and enhance AβP production [70,71]. Cu induces the dimerization and trafficking of APP from the ER to neurites. APP also regulates Fe homeostasis. APP mRNA possesses an iron responsive element (IRE). It means that the expression of APP is regulated by Fe, as well as ferritin (iron storage protein) [72]. In contrast, APP binds to ferroportin, which controls Fe efflux [73]. Therefore, APP is suggested to regulate the homeostasis of metals including Zn, Cu, and Fe.

### 3.3. Carnosine as an Anti-Crosslinker of AβP

Molecules that inhibit the oligomerization of AβP may be candidates for preventive AD therapeutics. Several compounds such as rifampicin, curcumin, and transthyretin have been reported to inhibit the oligomerization of AβP [52]. A small peptide composed of five amino acids, called a β-sheet breaker peptide, markedly blocks AβP oligomerization [74]. 

Carnosine has an anti-crosslinking ability, as shown in Figure 2, and inhibits the oligomerization of proteins such as α-crystalline [75]. Thus, *N*-acetyl carnosine has been used for the treatment of cataracts [76]. Increasing evidence indicates that carnosine inhibits the oligomerization of AβP and blocks its neurotoxicity [77,78]. Corona et al. demonstrated that orally administered carnosine inhibits the accumulation of AβP, and prevents learning deficits in a murine model of AD [20]. Moreover, histidine and carnosine are significantly reduced in the CSF of AD patients [79]. Therefore, carnosine may well play neuroprotective roles against AD.

## 4. Zinc, Carnosine, and Vascular Type of Dementia

### 4.1. Zinc and Ischemia-Induced Neuronal Death

VD accounts for about one-third of senile dementia cases in Japan. Its risk factors are high blood pressure and diabetes. The interruption of blood flow causes the oxygen-glucose deprivation and membrane depolarization after transient global ischemia or stroke [80]. Thereafter, an excess release of glutamate into synaptic clefts causes the overstimulation of the glutamate receptor and the entry of large quantities of Ca^2+^ into neurons, and triggers the death of pyramidal neurons in the hippocampus, which are crucial in memory formation. 

Zn plays a crucial role in the neuronal death after ischemia and the pathogenesis of VD [81,82]. Excess Zn can be neurotoxic in spite of its importance in the brain. The concentration of Zn in synaptic clefts is estimated to be 1–100 µM. However, a considerable amount of Zn (up to 300 µM) is co-released with glutamate into synaptic clefts during ischemic conditions [27]. Koh et al. demonstrated that Zn accumulates in apoptotic neurons in the hippocampus after ischemia [83]. The membrane-impermeable Zn chelator (calcium ethylene diamine tetraacetic acid (Ca-EDTA)) protects hippocampal neurons after ischemia and reduces infarct volume [84]. Zn can cause mitochondrial failure and oxidative stress [85]. 

### 4.2. Molecular Mechanism of Zn-Induced Neurotoxicity: GT1–7 Cells as an In Vitro Model System 

We investigated the molecular mechanism underlying Zn neurotoxicity and the protective mechanism of carnosine in immortalized hypothalamic neurons (GT1–7 cells). We found that Zn causes the death of GT1–7 cells in a dose-dependent manner [86]. The degenerated GT1–7 cells exhibited apoptotic characteristics including DNA fragmentation, as well as terminal deoxynucleotidyl transferase-mediated biotinylated uridine triphosphate (UTP) nick-end labeling (TUNEL)-positive. GT1–7 cells were revealed to be much more sensitive to Zn and exhibited much lower viability after Zn exposure compared with other neuronal cells, such as primary cultured rat hippocampal neurons, B-50 neuroblastoma cells, and PC-12 cells [87]. The GT1–7 cells were developed by Mellon et al. from mouse hypothalamic neurons by genetically targeted tumorigenesis [88]. The cells possess neuronal characteristics, such as the extension of neurites and expression of neuron-specific proteins or receptors. Meanwhile, the GT1–7 cells possess low levels of ionotropic glutamate receptors and are not subject to glutamate toxicity. These properties imply that the GT1–7 cell line is a good model system for the investigation of Zn-induced neurotoxicity.

Using GT1–7 cells, it was found that pyruvate, citrate, the antagonists of Ca^2+^ channels (nifedipine, conotoxine), and Al^3+^ blocked the Zn-induced death of GT1–7 cells [86,87,89]. We also found that intracellular Ca^2+^ levels ([Ca^2+^]_i_) are increased after exposure to Zn. The preadministration of Al^3+^, a Ca^2+^ channel blocker, inhibited the Zn-induced rise in [Ca^2+^]_i_ and attenuated Zn-induced neurotoxicity. Thus, it is highly possible that Ca^2+^ homeostasis is implicated in Zn neurotoxicity pathways.

### 4.3. Protective Substances against Zn-Induced Neuronal Death

Considering the significance of Zn in the pathogenesis of VD, it is highly possible that substances that inhibit Zn-induced neurotoxicity may become candidate drugs for the prevention or treatment of VD. Thus, we developed an assay system for screening such substances using GT1–7 cells and examined various agricultural products (such as fruits, vegetables, fish, and sea-products) [90]. Among the compounds tested, we found that the water-soluble extract of muscle tissue of Japanese eel (*Anguilla japonica*) exerted marked protective activity [91]. This activity was not diminished when the extract was boiled at 95 °C for 30 min. We separated the heated extract using HPLC and determined the structure of the active fraction as that of carnosine using liquid chromatography mass spectrometry (LC-MS). Moreover, we found the protective activities in the extract of mango fruits (*Mangifera indica*) and in the extract of round herring (*Etrumeus teres*), and determined the active fraction as pyruvate and histidine, respectively [92,93]. 

### 4.4. The Protective Roles of Carnosine

We investigated the neuroprotective mechanism of carnosine, and firstly focused on Zn translocation since carnosine can chelate Zn. First, we analysed intracellular Zn^2+^ levels ([Zn^2+^]_i_) in Zn-treated GT1–7 cells using Zn-specific fluorescent dye, ZnAF-2. However, neither carnosine nor anserine inhibited Zn influx into GT1–7 cells, whereas treatment with Ca-EDTA, a membrane impermeable chelator, decreased [Zn^2+^]_i_ [94]. Thus, it is plausible that carnosine did not act as a Zn chelator and protect neurons from Zn-induced neurotoxicity.

**Figure 5:**
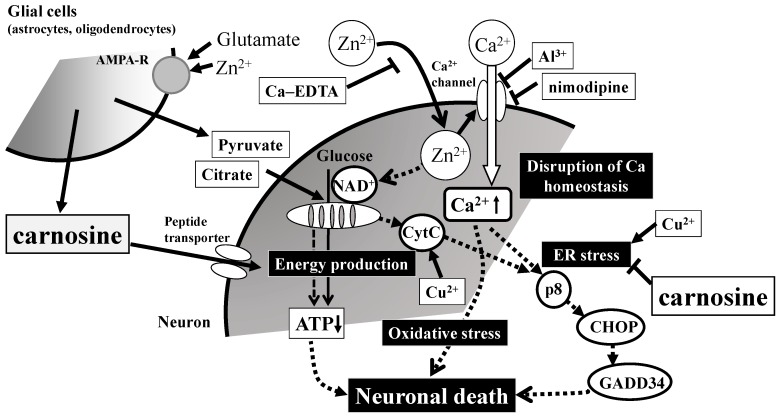
Possible molecular mechanisms underlying the protective effects of carnosine in preventing the neuronal death induced by Zn. Secreted excess amounts of Zn can translocate into cells and cause the disruption of Ca^2+^ homeostasis, energy failure in mitochondria, the induction of ER (reticulum) stress as well as oxidative stress, and apoptotic neuronal death. Carnosine is released into the synaptic cleft and is transported into cell bodies, where it can inhibit ER stress-related and/or Arc-related apoptotic pathways activated by Zn. Details are shown in the text. Ca-EDTA: calcium ethylene diamine tetraacetic acid; NAD^+^: nicotinamide adenine dinucleotide; CytC: cytochrome C; ATP: adenosine triphosphate; *CHOP*: CCAAT-enhancer-binding protein homologous protein; *GADD34*: growth-arrest- and DNA-damage-inducible gene 34. AMPA-R: AMPA-type glutamate receptor.

Second, we have analyzed the genetic changes induced by Zn, and our real-time polymerase chain reaction (RT-PCR) analysis revealed that Zn caused the upregulation of several genes, including metal-related genes (*MT-1*, *MT-2*, *ZnT-1*), ER-stress related genes (*GADD* (growth-arrest- and DNA-damage-inducible gene) 34, *GADD45*, *p8*, *CHOP* (CCAAT-enhancer-binding protein homologous protein)), and a Ca^2+^-related gene (*Arc*). Although carnosine is able to chelate Zn^2+^, we demonstrated that carnosine did not influence the intracellular concentration of Zn nor the Zn-induced upregulation of *MT-1* or *ZnT-1*. Meanwhile, carnosine was found to inhibit the upregulation of ER stress-related genes such as *GADD34*, *GADD45*, *CHOP*, Ca^2+^ homeostasis-related genes such as activity-regulated cytoskeleton-associated protein (*Arc)*. We also found that carnosine attenuated neurodegenerations induced by the ER-stressor such as thapsigargin and tunicamycin, and/or induced by hydrogen peroxide (H_2_O_2_). We demonstrated that anserine also attenuated Zn-induced neurotoxicity, as well as carnosine. Furthermore, we recently found that sub-lethal concentrations of Cu^2+^ strongly enhance Zn^2+^-induced neurotoxicity and the expression of ER stress-related genes [95]. The Cu^2+^ enhanced Zn^2+^-induced neurotoxicity was attenuated by pyruvate and thioredoxin-albumin fusion protein [96,97]. Taken together, we hypothesize the possible molecular mechanisms underlying Zn neurotoxicity and the action of carnosine (Figure 5). After exposure to Zn, intracellular Zn levels increase for at least 30 min. Chelators such as Ca-EDTA block this process. Zn leads to an increase in intracellular Ca^2+^ levels and then triggers ER stress. This process is inhibited by Al^3+^ and other Ca^2+^ channel blockers. Zn triggers the inhibition of the energy production machinery in the mitochondria and the production of oxidative stress. The energy substrates pyruvate and citrate prevent this process. The oxidative stress was attenuated by the thioredoxin-albumin fusion protein. Finally, these three processes trigger neurodegenerative pathways and lead to the neuronal death observed in VD. Carnosine, released from glial cells, enters neurons by peptide transporters and inhibits Zn-induced ER stress. Numerous in vivo studies have suggested that carnosine is protective against ischemia-induced neurodegeneration using experimental animals [98,99,100]. We have published a patent for carnosine to prevent or treat senile dementia based on the activities of carnosine [101].

## 5. Zinc, Carnosine, and Prion Diseases

### 5.1. Zinc, Copper and Prion Diseases

**Figure 6:**
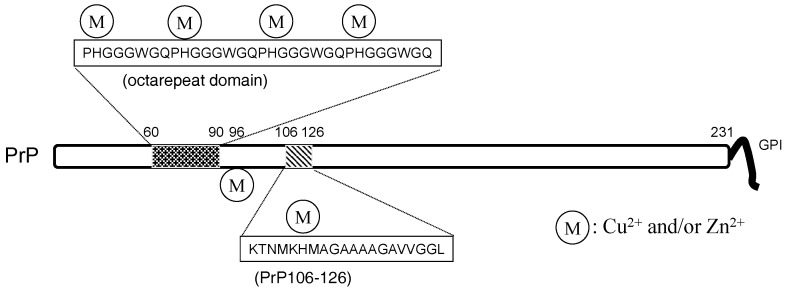
The structure of the prion protein. PrP: prion protein.

Zn and carnosine are also involved in prion diseases. Prion diseases include Creutzfeldt-Jakob disease (CJD), Gerstmann-Straussler-Scheinker syndrome (GSS), and Kuru disease in humans. They also include bovine spongiform encephalopathy (BSE) in cattle and scrapie in sheep [102]. Prion diseases are characterized by the spongiform degeneration of neurons and glial cells and the accumulation of amyloidogenic prion protein (PrP). The administration of pathogenic tissue causes the characteristic infections, and thus, prion diseases are also called transmissible spongiform encephalopathies. During the transmissible infections, a normal prion protein (PrP^C^) is converted to an abnormal scrapie-type isoform (PrP^Sc^). Although both of PrP^C^ and PrP^Sc^ have the same primary sequence, PrP^C^ differs from PrP^Sc^ in that PrP^Sc^ possesses a high content of β-sheet secondary structure compared to PrP^C^, and therefore has resistance to protease digestion. PrP^C^ is ubiquitously expressed throughout the entire body (Figure 6). It is speculated that the misfolded PrP^Sc^ administrated from contaminated food induces normal PrP^C^ molecules in the brain to misfold and aggregate. 

Therefore, prion diseases are considered to be protein-misfolding diseases (conformational diseases), namely, the conformational change from PrP^C^ to PrP^Sc^ is crucial for the pathogenesis of prion diseases [103]. These features are similar to AD. PrP^C^ is reported to be a metalloprotein and regulates metal homeostasis [41]. PrP^C^ is a 30–35-kDa glycoprotein and contains 208 amino acid residues. In its N-terminal, PrP^C^ possesses an octa-repeat domain composed of multiple tandem copies of the eight-residue sequence –PHGGGWGQ– The octa-repeat domain can bind four metal ions (including Cu^2+^, Zn^2+^, and other divalent ions), and two other histidine residues, His^96^ and His^111^, can bind two metal ions [104]. The levels of Cu in the brains of PrP-knockout mice were decreased compared with wild-type mice [105]. PrP^C^ possesses protective roles against oxidative stress as Cu/Zn superoxide dismutase (Cu/Zn SOD) [106]. 

Zn^2+^ also binds PrP, and can influence Cu binding to PrP^C^. Moreover, prion genes and genes encoding ZIP transporters possess evolutionary sequence similarities [107]. Watt et al. hypothesized that PrP^C^ acts as a ‘Zn sensor’ and facilitates the uptake of Zn into neurons by binding with AMPA receptors [108]. 

### 5.2. Carnosine and PrP^Sc^-Induced Neurotoxicity

Normal PrP^C^ has regulatory and neuroprotective functions such as regulating Cu homeostasis and antioxidant. Thus, loss of the protective functions by converting to PrP^Sc^ will lead to neurodegeneration. Meanwhile, PrP^Sc^ is neurotoxic. Since PrP^Sc^ has strongly infectious characteristics, using a full-length prion protein is difficult [109]. Thus, we and other researchers have used synthetic fragment peptides of PrP (PrP106–126) in the study of PrP^Sc^ neurotoxicity, owing to its similar characteristics with PrP^Sc^ such as β-sheet formation, neurotoxicity, and metal-binding ability. 

Our results using primary cultured rat hippocampal neurons and the thioflavin T (ThT) fluorescence method exhibited that PrP106–126 forms β-sheet structures during the “aging” process (incubation at 37 °C for several days), and that aged PrP106–126 causes significant neurotoxicity. These characteristics are quite similar to AβP [22]. Either Zn^2+^ or Cu^2+^ significantly attenuates PrP106–126 neurotoxicity. Furthermore, either Zn^2+^ or Cu^2+^ inhibits the oligomerization of PrP106–126 during the aging process, observed by thioflavin T fluorescence and atomic force microscopy observation.

**Figure 7:**
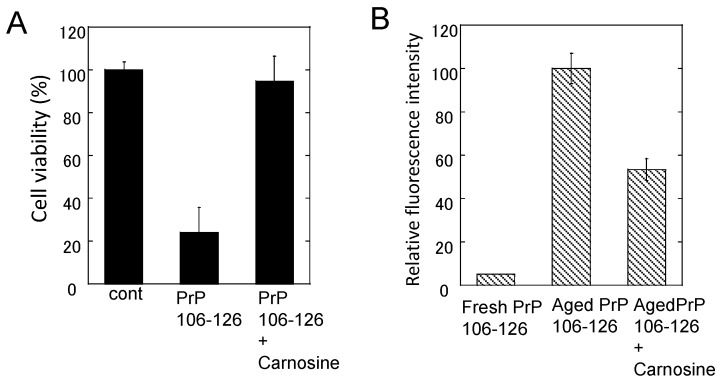
Effects of carnosine on the neurotoxicity and the conformational changes of PrP106–126. (**A**) Effects of carnosine on the neurotoxicity of PrP106–126. The viability of cultured rat hippocampal neurons was analyzed using the lactate dehydrogenase (LDH) method after three days of exposure to 50 μM PrP106–126 aged alone or with 1 mM carnosine. Data are presented as mean ± S.E.M. (*n* = 6); (**B**) Effects of carnosine on the thioflavin T (ThT) fluorescence of PrP106–126. The ThT fluorescence (ex. 490 nm, em. 520 nm) of 25 μM of fresh PrP106–126 or aged PrP106–126 with 1 mM carnosine was analyzed. Data are presented as mean ± S.E.M. (*n* = 7).

Although chelators such as clioquinol and deferoxamine did not influence PrP106–126 neurotoxicity, we found that carnosine attenuates the neurotoxicity of PrP106–126 and inhibits oligomerization (Figure 7). Therefore, it is possible that carnosine acts as an anti-crosslinker of PrP106–126. Carnosine also inhibits the oligomerization of α-synuclein, which is a major player in DLB and Parkinson’s disease [110], and α-crystalline in lens [111]. In this respect, the anti-crosslinking activity of carnosine is important for the protective functions of carnosine against these diseases.

## 6. Crosstalk of Metals and Amyloidogenic Proteins at Synapse

### 6.1. Colocalization of APP and PrP at Synapse

Synapses are small but critical nodes for information processing and memory formation in neural networks. Since synaptic plasticity is essential to memory formation, synaptic degenerations are primary observed in many neurodegenerative diseases. As discussed in the previous sections, metals (Zn^2+^ or Cu^2+^) as well as neurotransmitters are co-released from synaptic vesicles into synaptic clefts. They bind to receptors at postsynaptic densities. 

Two amyloidogenic proteins, APP and PrP^C^, are localized in the synapse, and play essential roles in the regulation of metal homeostasis. APP is in the presynaptic region and AβP is secreted into synaptic clefts in the presence of neuronal stimuli [112]. PrP^C^ is coupled to glutamate receptors in the postsynaptic membranes [113]. Considering the short distance across the synaptic cleft (approximately 20 nm), APP can interact with PrP^C^ in this small compartment surrounded by a considerable amount of Zn^2+^ and Cu^2+^. Indeed, PrP^C^ reportedly binds to AβP oligomers and attenuates its neurotoxicity.

Given that the homeostasis of metals regulated by APP and PrP^C^ is disrupted, it triggers the degeneration of synapses, causing neurodegeneration and finally leading to the pathogenesis of these diseases. ZnT-1 is also localized in postsynaptic membranes and regulates Zn homeostasis by enhancing Zn efflux to the extracellular compartment [114]. ZnT-1 also regulates the activity of NMDA-type glutamate receptors. Meanwhile, PrP^C^ controls Zn^2+^ influx into cells as an analogue of ZIP transporters, with AMPA-type glutamate receptors regulating synaptic Zn^2+^ levels. MT-3, brain specific metallothionein, may also regulate Zn homeostasis at synapses [115]. In addition, MT-3 is decreased in the brains of patients with AD [116]. 

### 6.2. Carnosine: A Regulator of Zn and Cu in the Synapse

**Figure 8:**
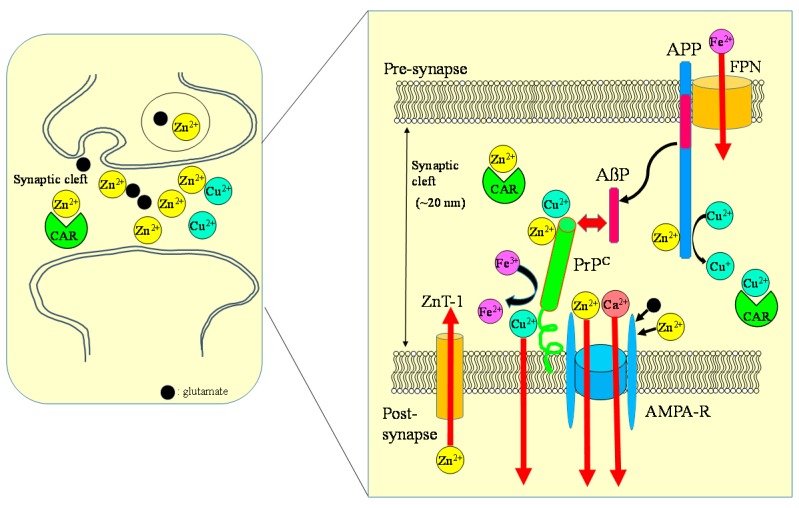
Crosstalk between carnosine, metals, APP, and PrP at the synapse. Zn, Cu, and glutamate accumulate in synaptic vesicles and are released into synaptic clefts during neuronal excitation. Under normal physiological conditions, APP binds Cu and regulates Cu levels by reducing Cu^2+^ to Cu^+^. The normal prion protein isoform PrP^C^ binds to Cu at its N-terminal domain and regulates synaptic Cu levels. It is possible that PrP^C^ provides Cu to APP or to NMDA-type glutamate receptors, thereby influencing the production of AβP or neuronal excitability. Both APP and PrP^C^ reportedly attenuate Cu-induced toxicity. PrP^C^ also controls Zn^2+^ influx into the cells as an analogue of ZIP transporters, with AMPA-type glutamate receptors regulating synaptic Zn^2+^ levels. ZnT-1 is localized to postsynaptic membranes that express NMDA-type glutamate receptors and regulates Zn homeostasis. APP binds ferroportin (FPN) and regulates Fe^2+^ efflux. By contrast, PrP^C^ acts as ferrireductase to regulate the Fe^2+^/Fe^3+^ ratio in synapses. The Fe^2+^ ions are transferred to enzymes, including neurotransmitter synthetases. Fe levels also regulate the expression of APP. It is plausible that carnosine binds to Zn^2+^ and Cu^2+^ and regulates homeostasis in these metals. ZnT-1: zinc transporter 1; NMDA-R: NMDA-type glutamate receptor; MT-3: metallothionein 3; CAR: carnosine.

Carnosine is another important contributor to regulating metal homeostasis in the synapse. The concentration of carnosine in the olfactory bulb is as high as 0.5 mM [18]. Carnosine and homocarnosine is synthesized in glial cells, and carnosine-like immunoreactivity was observed in astrocytes or oligodendrocites [117]. Carnosine is reported to be secreted into the synaptic cleft from oligodendrocytes by glutamate in a Zn-dependent manner [36,118]. Therefore, carnosine is suggested to contribute the availability of Zn at the synapse and to control Zn homeostasis and, therefore, provides wide protection against various neurodegenerative disorders. Figure 8 exhibits a hypothetical scheme showing interactions between carnosine, APP, PrP, Zn, and Cu at the synapse.

## 7. Carnosine in Foodstuffs

Considering the beneficial and neuroprotective roles of carnosine, dietary supplementation with carnosine may become important for the prevention of various neurodegenerative diseases. The supplementation with carnosine/anserine improves the cognitive decline of AD model mice [119], or of elderly people [120]. Orally administered carnosine can cause increased carnosine levels in the brain [121]. Moreover, the amount of carnosine is age-dependently decreased [122]. 

**Figure 9:**
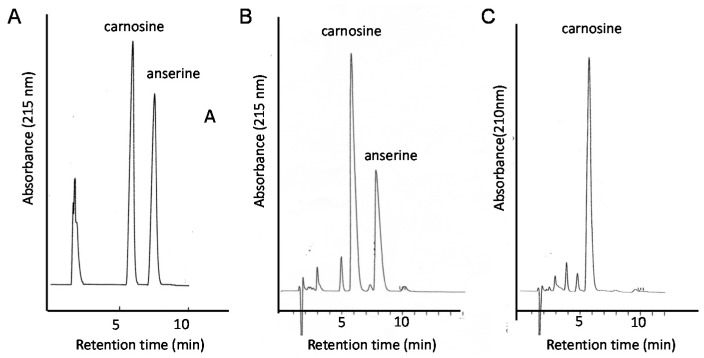
Typical chromatograms of the food extracts examined in this study. Standard solutions carnosine and anserine (**A**); extract of chicken breast (**B**), or extract of pork (**C**) were applied to the HPLC system equipped with a carbon column. Eluent: 0.05% TFA and 7% CH_3_CN; flow rate: 1.0 mL/min. The absorbance at 215 nm was observed.

Therefore, the quantitative analysis of carnosine in foods is essential for developing supplementation therapy. For this purpose, we established a convenient system for the analysis of carnosine and anserine in various foods using HPLC [123,124]. However, it is difficult to separate carnosine and anserine by performing reversed phase HPLC with a conventional octadecylsilyl (ODS) column used in ordinary peptide analysis, since carnosine and its analogues are highly hydrophilic and not retained in an ODS column. Thus, we used a carbon column (Hypercarb™ column; Thermo Electron Corp., Waltham, MA, USA) containing porous graphite carbon. The typical chromatograms of standard carnosine and anserine are shown in Figure 9A. Under these conditions, carnosine appeared at 5.7 min and anserine at 7.3 min. We used conventional UV spectroscopy for measuring the absorbance at 215 nm to detect carnosine and anserine. The typical chromatograms of the water extract of chicken breast and the pork shoulder after being heated at 95 °C for 30 min to reduce and remove proteins are shown in Figure 9B,C. The recovery rate of carnosine was determined to be 98.8 ± 6.6% and that of anserine was 99.4 ± 1.8% after this simple pretreatment. 

**Figure 10:**
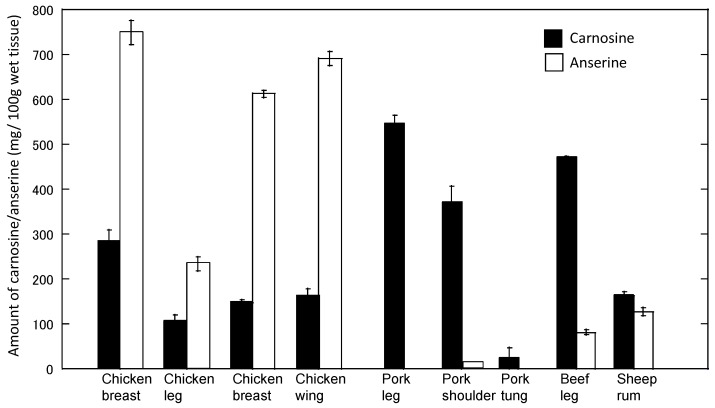
Levels of carnosine and anserine in various foods. The amount of carnosine and anserine of water-soluble extracts of various foods was analyzed by HPLC. Data are expressed as mean ± S.E.M. (*n* = 3).

Using this method, the level of carnosine and anserine in food extracts was examined (Figure 10). Our results suggest that relatively high concentrations of carnosine exist in muscles. For example, 1 g of chicken muscle (breast) contained 2.16 ± 0.67 mg/g (i.e., approximately 1 mM) of carnosine. The concentrations of carnosine and anserine were different among species and varied in various regions. These results coincide with previous studies [18].

Furthermore, we analyzed the amount of carnosine in the muscle of thoroughbred horses and found that the gluteus medius exhibited the highest concentration of carnosine among five muscle tissues (flexor capri radius, triceps branchii, masseter, gluteus medius, sternocleidomastoid). Considering that the gluteus medius is abundant in Type IIa muscles and mainly used in high intensity exercise, carnosine may play essential roles in high intensity exercise. Indeed, the supplementation of β-alanine reportedly increases muscle carnosine concentrations and improves exercise performance, as concluded by the International Society of Sports Nutrition [125].

## 8. Conclusions and Future Perspectives

Zn plays critical roles in the pathogenesis of neurodegenerative diseases, such as AD, VD, and prion diseases. Although Zn acts as an enhancer of neurotoxicity as well as a protector in AD and VD, it is possible that carnosine, a Zn chelator, acts as a neuroprotector in these diseases owing to its numerous beneficial characteristics including anti-ER stress, antioxidant, and anti-crosslink activities. Additionally, the properties of carnosine such as being water-soluble, heat-inactive, and nontoxic make it a good neuroprotective nutrient beneficial for health. Considering the beneficial characteristics of carnosine, dietary supplementation with carnosine or its component may become useful for health, as carnosine in food plays a critical role in the regulation of Zn homeostasis and the prevention of neurodegenerative diseases. Further research about the molecular mechanism of carnosine in preventing neurotoxicity is required.

## Abbreviations

AD	Alzheimer’s disease
AMPA	amino-3-hydroxy-5-methyl-4-isoxazolepropionic acid
CSF	cerebrospinal fluid
EAR	estimated average
ER	endoplasmic reticulum
GABA	γ-aminobutyric acid
HPLC	high performance liquid chromatography
NMDA	*N*-methyl-d-aspartate
VD	vascular type of senile dementia
ZIP	Zrt-, Irt-like protein
